# Safety of Deep Enteroscopy and Capsule Endoscopy in LVAD Patients: Case Report and Literature Review

**DOI:** 10.14740/gr666w

**Published:** 2015-12-31

**Authors:** Wilson Tak-Yu Kwong, Michelle Pearlman, Denise Kalmaz

**Affiliations:** a Department of Medicine, University of California San Diego, La Jolla, CA, USA; b Division of Gastroenterology, University of California San Diego, La Jolla, CA, USA

**Keywords:** Double balloon enteroscopy, Overtube-assisted enteroscopy, LVAD, Left ventricular assist device, GI bleeding

## Abstract

Patients with a left ventricular assist device (LVAD) have increased risk of gastrointestinal (GI) bleeding. They are prone to develop angiodysplasia of the small intestine, and have a higher risk of bleeding as these patients are all required to be on permanent therapeutic anticoagulation. Here we report a case of a critically ill 55-year-old male on pressors and inotropes with an LVAD, who successfully underwent an antegrade double balloon enteroscopy (DBE).

## Introduction

Patients with a left ventricular assist device (LVAD) are at increased risk for gastrointestinal (GI) bleeding with 20% of patients experiencing at least one episode [1, 2]. Approximately a quarter of these episodes of overt GI bleeding originate from the small intestine [3]. This is likely an underestimate as studies consistently show a high rate without identifiable bleeding source. Despite high rates of small intestinal bleeding, endoscopic evaluation of the small bowel with capsule endoscopy is rarely performed, while deep enteroscopy (spiral enteroscopy, single balloon enteroscopy, and double balloon enteroscopy) is even less so. The slow adoption of small bowel endoscopy in LVAD patients is likely related to uncertainty regarding safety of these procedures in a population with severe cardiac comorbidity with a new, evolving technology. We present an illustrative case with the second description of double balloon enteroscopy (DBE) in an LVAD patient, and review the literature regarding the safety of deep enteroscopy and capsule endoscopy in patients with an LVAD.

## Case Report

**Figure 1:**
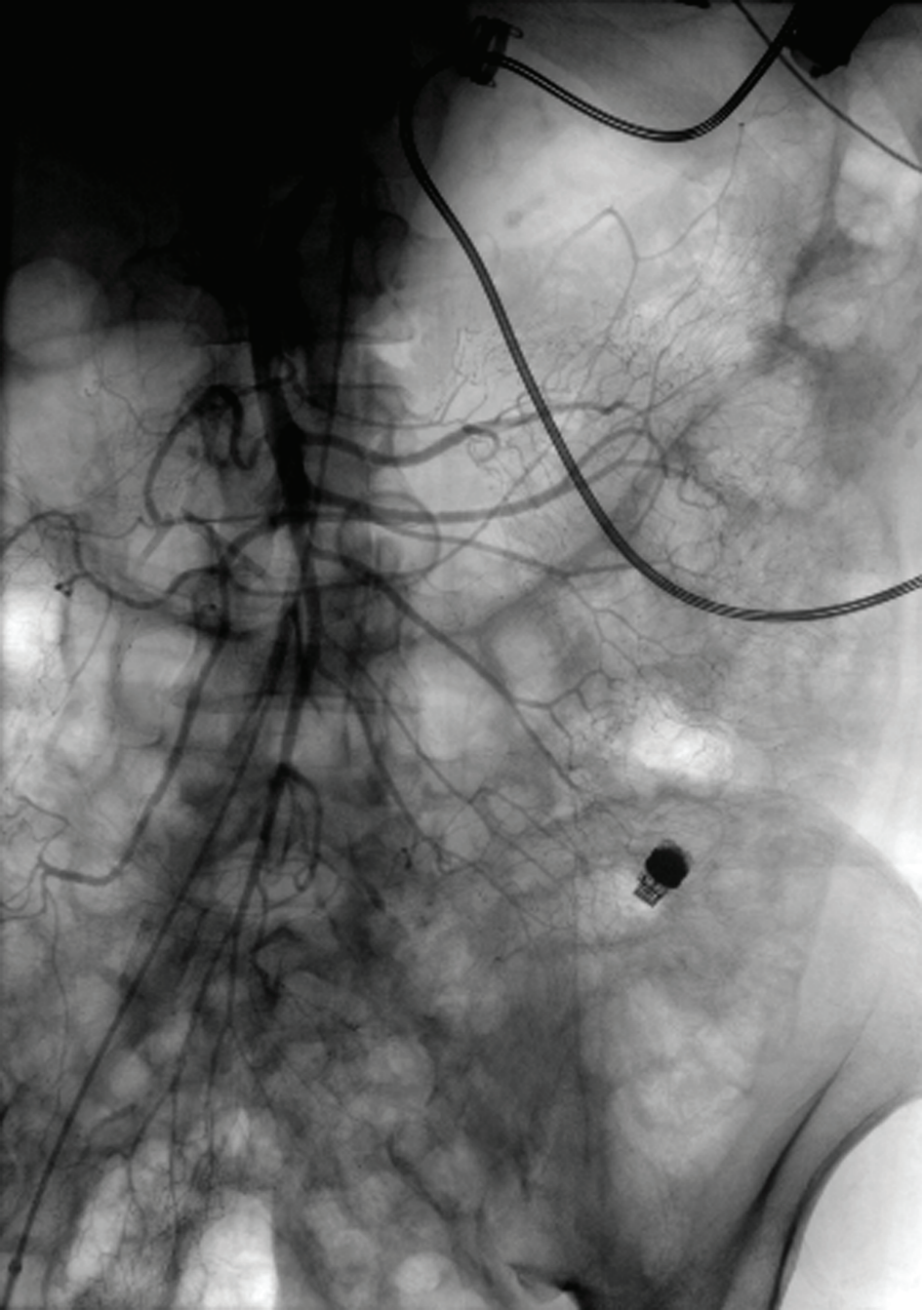
Angiogram of the superior mesenteric artery without extravasation of contrast. Notice the LVAD with driveline in the left upper quadrant and the PillcamSB in the left lower quadrant.

**Figure 2:**
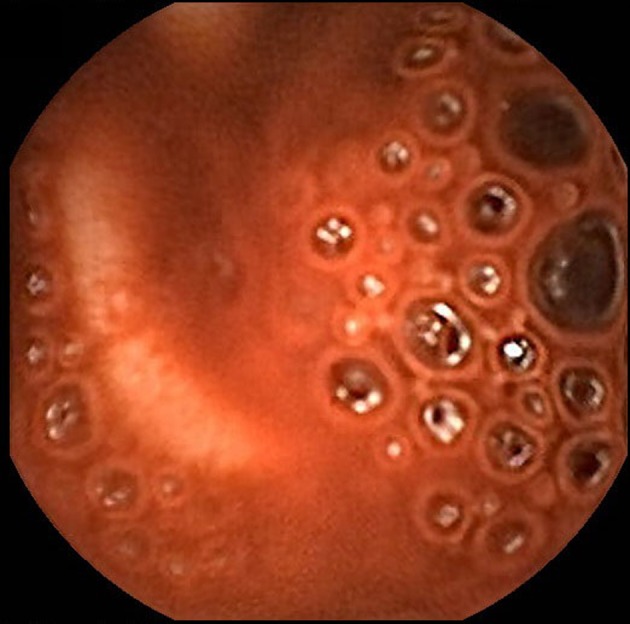
Capsule endoscopy demonstrates active bleeding in the proximal jejunum but blood obscures identification of the bleeding source.

A 55-year-old male with dilated cardiomyopathy, atrial fibrillation, internal cardiac defibrillator, end-stage renal disease on hemodialysis, and iron deficiency anemia was transferred to our institution for LVAD placement. Esophagogastroduodenoscopy (EGD) and colonoscopy performed 4 months prior to LVAD placement to evaluate iron deficiency anemia were unremarkable. The patient remained hospitalized after HeartWare (Framingham, MA, USA) LVAD placement due to a complicated course including fungemia, ventricular arrhythmias, and aspiration pneumonia resulting in cardiac arrest and intubation from which he subsequently recovered. Three months after LVAD placement, the patient was transferred to the intensive care unit (ICU) for septic shock and required norepinephrine and dobutamine infusions for vasopressor and inotropic support. The following day, the patient developed maroon colored stools in the setting of aspirin and therapeutic anticoagulation with decrease in hemoglobin (Hgb) from 9 to 6 g/dL. He was transfused six units of red blood cells (RBCs). Urgent EGD and colonoscopy without bowel preparation demonstrated bilious fluid in the duodenum and blood throughout the terminal ileum and colon; however, the bleeding source could not be identified. Given suspicion for a small bowel source of bleeding, a PillcamSB (Given Imaging, Yoqneam, Israel) capsule study was performed. The patient continued to demonstrate signs of active GI bleeding and a tagged RBC scan was performed concurrently, revealing active blood loss from the small bowel. Mesenteric angiogram (Fig. 1) of the celiac and superior mesenteric arteries did not reveal extravasation of contrast. The capsule study demonstrated active bleeding in the proximal jejunum, although the bleeding source was obscured by blood (Fig. 2, Supplementary Video 1, www.gastrores.org). Push enteroscopy reached the proximal jejunum without signs of blood or a source of bleeding. The patient was subsequently intubated for worsening respiratory status and continued to require dobutamine and norepinephrine for hypotension. After discussion with cardiology, anesthesia, and cardiothoracic surgery, the decision was made to perform antegrade DBE given ongoing requirement for anticoagulation in setting of an LVAD. DBE was performed at the bedside in the cardiac ICU. The double balloon enteroscope (Fujinon, Tokyo, Japan) was advanced 250 cm past the ligament of Treitz without any signs of blood or potential bleeding source. The suspected bleeding source was a proximal jejunal Dieulafoy’s lesion given the absence of mucosal lesions or angiodysplasia visualized on DBE. The patient tolerated the procedure without complication and his episode of bleeding ceased.

## Discussion

Bleeding in patients with LVADs is likely to become a more frequently encountered problem given their increasing usage as destination therapy and bridge to transplantation. Evaluation of the small bowel appears to be of particular importance among LVAD patients given that there is increased small bowel angiodysplasia and up to 65% of patients have unidentified source of bleeding despite upper and lower endoscopy [4]. The high rates of unidentified sources of bleeding may be partly due to infrequent usage of capsule endoscopy and deep enteroscopy including spiral enteroscopy, single balloon enteroscopy (SBE), and DBE (Table 1) [2-15]. The infrequent usage of these modalities to evaluate the small bowel may be attributed to uncertainty regarding the safety of these procedures in this patient population.

**Table 1:** Summary of Published Reports of Capsule Endoscopy and Small Bowel Enteroscopy in LVAD Patients

Publication author, year	Capsule study	Enteroscopy	Direct complications of capsule study or enteroscopy
Girelli et al, 2006 [9]	1		None
Seow and Zimmerman, 2006 [10]	1	One push	None
Fenkel et al, 2007 [11]	1		None
Daas et al, 2008 [12]	1	One push	None
Bechtel et al, 2010 [13]	1		None
Decker et al, 2010 [5]	1	One DBE	None
Stern et al, 2010 [4]	3		None
Demirozu et al, 2011 [14]		Two push	None
Elmunzer et al, 2011 [6]	13	Four SBE/DBE; three push	None
Huang et al, 2012 [15]	1		None
Kushnir et al, 2012 [2]	5	One push	Duodenal ulcer perforation after three episodes of electrocautery
Harris et al, 2013 [8]	14		Two patients with brief interruptions (< 2 min) in capsule imaging
Sarosiek et al, 2013 [7]		Thirteen spiral/SBE/DBE	None
Shrode et al, 2014 [3]	20	Ten SBE/DBE	None
Total	62	Eight push; 28 spiral/SBE/DBE	One duodenal ulcer perforation Two disruptions of capsule imaging

DBE: double balloon enteroscopy; min: minutes; push: push enteroscopy; SBE: single balloon enteroscopy; spiral: spiral enteroscopy.

These procedures theoretically carry a higher risk compared to endoscopy and colonoscopy given the prolonged procedure and sedation time, repeated episodes of scope advancement and withdrawal with resultant pressure and mobilization of the small bowel in the upper abdomen. The LVAD driveline, which connects the LVAD to the external controller and power source, travels through the upper abdomen creating the potential of disrupting the driveline during deep enteroscopy. There is also concern that a capsule study may interfere with LVAD functioning and vice versa.

Review of the literature reveals that 62 capsule studies and 28 deep enteroscopies have been performed in LVAD patients. There has been one prior documented DBE performed in an LVAD patient [5] while three other studies did not distinguish spiral enteroscopy, SBE, and DBE [3, 6, 7]. The current case is the first DBE to be performed in a critically ill patient demonstrating it is possible to perform DBE safely even in the sickest of patients. Despite the limited number of reported LVAD patients undergoing capsule endoscopy and deep enteroscopy, these procedures appear to be quite safe in the LVAD population. The only major complication was a case of duodenal ulcer perforation after three attempts at cauterization. This complication is the result of attempted intervention rather than the enteroscopy itself. In addition, there are no reports of capsule endoscopy interfering with LVAD function, and only two reports of minimal interruptions in capsule image acquisition [8] which may not necessarily be the result of the LVAD’s presence. There are also no reports to date of deep enteroscopy disrupting the LVAD circuit or the driveline. With increasing experience demonstrating safety, capsule endoscopy and balloon assisted enteroscopy can become more widely utilized in the LVAD population which has particularly high rates of small bowel bleeding and a requirement for permanent anticoagulation.
